# 75202 The New Normal: A Virtual Summer Foundations in Research

**DOI:** 10.1017/cts.2021.563

**Published:** 2021-03-30

**Authors:** Adriana Morales Gomez, Kit Knier, Joanna Yang Yowler, Chris Pierret, Linda M. Scholl

**Affiliations:** 1 Mayo Clinic Graduate School of Biomedical Sciences, Mayo Clinic, Rochester, MN, USA; 2 Mayo Clinic Medical Scientist Training Program, Mayo Clinic, Rochester, MN, USA; 3 Department of Research, Mayo Clinic, Jacksonville, FL, USA; 4 Department of Biochemistry and Molecular Biology, Mayo Clinic, Rochester, MN, USA; 5 Office of Applied Scholarship and Education Science, Mayo Clinic College of Medicine, Mayo Clinic, Rochester, MN, USA

## Abstract

ABSTRACT IMPACT: The Summer Foundation on Research gave undergraduate students the opportunity to do research despite the new normal - COVID-19 pandemic. OBJECTIVES/GOALS: The COVID-19 pandemic prevented domestic and international undergraduate students from attending in-person Mayo Clinic Summer Undergraduate Research Programs. Mayo decided to redesign this program as a virtual, 4-week Summer Foundations in Research (SFIR) program. The goal of this program was to give students a scientific research experience. METHODS/STUDY POPULATION: The SFIR included an Introduction to Experimental Design, Dialogue methodology for communicating science, scientific mentoring, asynchronous online modules and a Resiliency component. Evaluations of the program were undertaken to gather feedback for program improvement and to assess the educational and mental health impact on participants. These evaluations asked student to rate each section of the program. Additionally, students were encouraged to provide their own comments and feedback. Statistical analysis of quantitative data was performed using excel. The qualitative data was studied using the identification, analysis and interpretation of patterns method per the student’s comments on each of the questions addressed in the survey. RESULTS/ANTICIPATED RESULTS: These evaluations revealed positive outcomes across program components: 66% of the participants found the Resiliency component extremely worthwhile, 80% of participants liked the experimental design and 70% liked the educational courses. Qualitative data showed that mentor/mentee interactions were highly valued, and both participants and faculty suggested increasing the amount of time devoted to these interactions. Small group discussions gave students the opportunity to get to know other peers and encouraged further discussions about science and the community. Participants suggested minor improvements to the program, such as re-creating the online modules specific for undergraduate students, increasing 1-to-1 and small group’s discussion, and increasing the length of the program. DISCUSSION/SIGNIFICANCE OF FINDINGS: Despite the quick pivot of the SFIR program, the re-design and new format supported the development of participants’ resilience skills and training as future scientists during a particularly challenging time. Mayo is committed to continuing this program as an early step in a pathway to careers in research.

